# Blood-Letting in Pneumonia

**Published:** 1896-02

**Authors:** A. N. Perkins

**Affiliations:** Sabine Pass


For the Texas Medical Journal.

### BLOOD-LETTING IN PNEUMONIA.

BY A. N. PERKINS, M. D., SABINE PASS.

FORTY years ago it was as much the custom of the physician to carry a lancet in his pocket as it is now for him to carry a thermometer. It was considered an essential part of his paraphernalia, and no physician thought of entering the field of a general practice without the little instrument in his pocket. It was the mystic wand by which he controlled a tumultous heart and reduced a consuming temperature to the limit of safety. The abstraction of blood was considered a necessity in all inflammatory diseases, and for a physician to lose a case of pneumonia without having bled him in the course of his treatment, would have aroused the neighborhood and greatly endangered his future prospects.

I recollect as vividly as if it were yesterday, the first case of pneumonia I ever treated. The patient was a young farmer 23 or 24 years old. He had a wife and baby. The night before I saw him he had a severe chill. When I arrived at his bedside his fever was high. He complained of severe pain in head and breast, with a constant disposition to cough, expectorating a bloody mucus. He was propped up in bed on account of difficulty in breathing. I immediately corded his arm and bled him until he complained of being faint and sick. I then stopped the flow of blood and prepared for him a tea of tart emetic, which I gave in doses sufficient to keep his skin soft and moist. In the evening I gave him calomel with Dover's powders sufficient to give him ease and rest. The next day I found him a great deal better. With quinine, counter-irritation, etc., he was soon convalescent. It is a question with me, whether the most accepted modern treatment of pneumonia unaccompanied with venesection, will relieve a similar case of pneumonia as quickly, and with as little injury to the affected organs.

There is no remedy that will relieve the strain and muscular tension like blood-letting. It at once lessens the momentum of the heart that is driving the blood like a forcing pump into the engorged lung and threatening over distension and paralysis of capillary vessels.

The condition of the patient is often too perilous and critical to wait for the effects of the remedies used at the present day as substitutes of the venesection. Relief must be given at once or the respiratory organ will be so deluged with effusions and consolidated with exudates that the patient will cease to breathe. Every physician in general practice meets with such cases, and I am sorry to say every one of them proves fatal unless treatment by a bold and independent practitioner who comprehends the condition and danger of his patients, and dares to do his duty in the face of prejudice and denunciation. Delay and vacilation in such cases means death. The action of drugs is too slow, there is no time to await for their effects.

There is no way to save such cases except to open a vein and relieve arterial tension and pulmonary engorgement. The fear of debility in such cases is a bug bear, and has caused the premature death of many useful men and women. Venesection in such cases, strengthens instead of weakens, by relieving the engorgement and giving relief to the over burdened heart.

There is no doubt but that this remedy, powerful as it is for good or evil, was at one time greatly abused. This is no argument against its proper use. I am of the opinion, the day is near at hand when it will again be considered an important remedy in the treatment of pneumonia. Of course blood-letting is not necessary in every case of pneumonia. On the contrary, much the larger proportion of cases do not call for it. But I repeat there are cases, and every practicing physician is liable to meet with them, that can not be saved without free and copious bleeding in the early stages. Without blood-letting they will surely die, with it they may be saved. I conclude therefore, by saying, every physicians should carry a lancet, and should not hesitate to use it when indications call for it.

